# Supplementary material for: Spatial transcriptomics using combinatorial fluorescence spectral and lifetime encoding, imaging and analysis
Source: Nat Commun. 2022 Jan 10;13:169. doi: 10.1038/s41467-021-27798-0 (PMC8748653; doi:10.1038/s41467-021-27798-0)
Supplement: Supplementary file 1 — Supplementary Information [file 41467_2021_27798_MOESM1_ESM.pdf]

Spatial transcriptomics using combinatorial fluorescence spectral and lifetime encoding, imaging and analysis

Supplementary Material

Tam Vu, Alexander Vallmitjana, Joshua Gu, Kieu La, Qi Xu, Jesus Flores, Jan Zimak, Jessica Shiu, Linzi Hosohama, Jie Wu, Christopher Douglas, Marian L. Waterman, Anand Ganesan, Per Niklas Hedde, Enrico Gratton, and Weian Zhao

Supplementary Tables

Table 1. List of Fluorophores Used

Fluorophore	Detection Channel	Excitation Max (nm)	Emission Max (nm)	Extinction Coefficient (M-1cm-1)	Quantum Yield	Brightness	Lifetime (ns)
ALEXA 647	1	650	668	270,000	0.33	89,100	1.04
ATTO 647N	1	646	664	150,000	0.65	97,500	3.5
ATTO 590	2	593	622	120,000	0.8	96,000	3.7
ATTO 565	3	564	590	120000	0.9	108,000	4
ATTO 488	4	500	520	90,000	0.8	72,000	4.1

Table 2. List of Genes Used and their Assigned Fluorophore Combination

Gene	Fluorophore 1	Fluorophore 2	No. of Probes per Gene	Present in Channel(s)	Used in Figure(s)
NCOA3	ATTO 565	ATTO 590	80	2, 3	4
POLR2A	ATTO 488	ALEXA 647	80	1, 4	4
MTOR	ATTO 565	ATTO 647	80	1, 3	4
MKI67	ATTO 647	ALEXA 647	80	1	4
BRCA1	ATTO 590	ALEXA 647	80	1, 2	4
NCOA2	ATTO 590	ATTO 647	80	1, 2	4
BRCA2	ATTO 488	ATTO 565	80	3, 4	4, 5
CENPF	ATTO 488	ATTO 590	80	2, 4	4, 5
CKAP5	ATTO 565	ALEXA 647	80	1, 3	4, 5
NCOA1	ATTO 488	ATTO 647	80	1, 4	4, 5

Supplementary Note 1. Phasor Transform Expressions

The first-order phasor transform of the lifetime intensity photon histogram $I(t)$ is defined as:

$$S = \frac{\int_0^T I(t)\sin(\omega t)dt}{\int_0^T I(t)dt} \tag{1}$$

$$G = \frac{\int_0^T I(t)\cos(\omega t)\,dt}{\int_0^T I(t)\,dt} \tag{2}$$

Where T is the period between excitation pulses (or modulation period) and $\omega = \frac{2\pi}{T}$ is the pulsation frequency such that the period of the trigonometric functions matches the excitation period T.

The first-order phasor transform of the spectral intensity photon histogram $I(\lambda)$ is defined as:

$$S = \frac{\int_{\lambda_0}^{\lambda_f} I(\lambda)\sin(\omega\lambda - \omega\lambda_0)\ d\lambda}{\int_{\lambda_0}^{\lambda_f} I(\lambda)\,d\lambda} \tag{3}$$

$$G = \frac{\int_{\lambda_0}^{\lambda_f} I(\lambda)\cos(\omega\lambda - \omega\lambda_0)\ d\lambda}{\int_{\lambda_0}^{\lambda_f} I(\lambda)\,d\lambda} \tag{4}$$

Where λ_0 and λ_f are the limits of the spectral band of the detector and $\omega = \frac{2\pi}{\lambda_f - \lambda_0}$ is the pulsation frequency such that the period of the trigonometric functions matches the spectral bandwidth $[\lambda_0\ \lambda_f]$.

Supplementary Figures

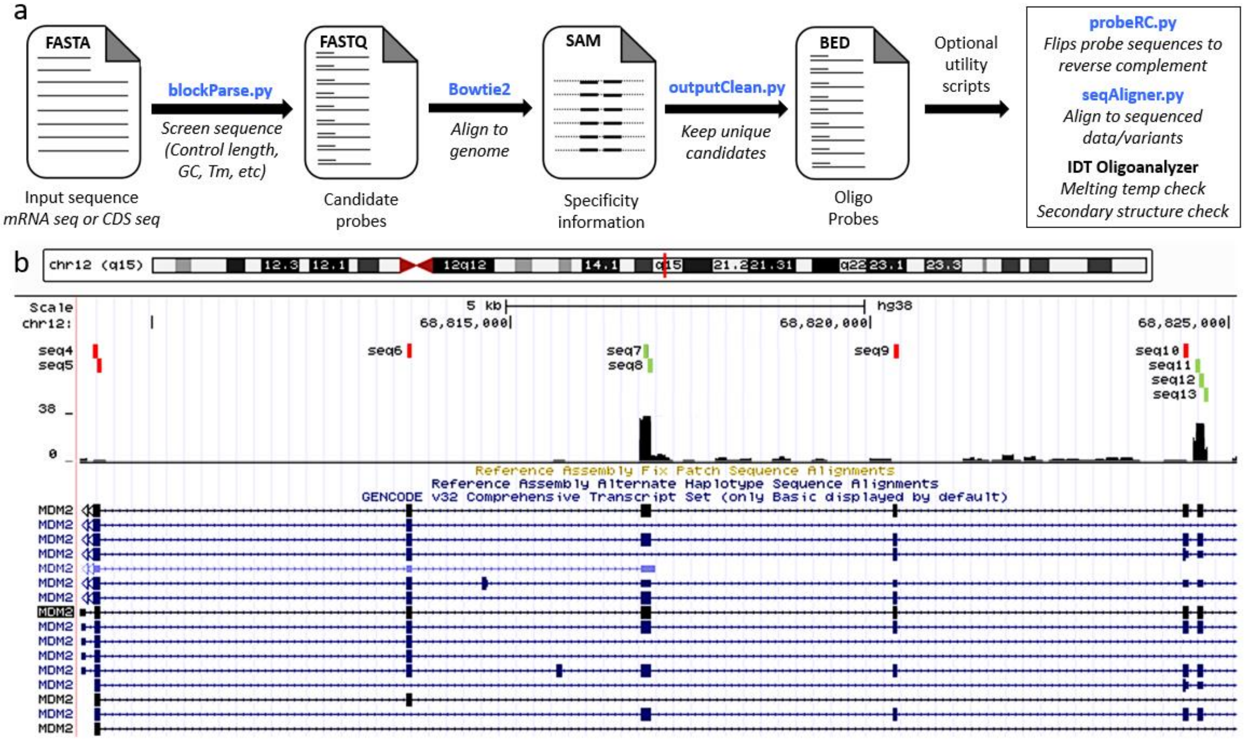

Supplementary Fig. 1. Schematic overview of probe design process. a) *A modified version of Oligominer was used to design primary probes.* ***b)*** *In the seqAligner.py script, the probes (labeled "seq#") go through BLAT and are aligned to the RNA sequencing data, as seen on the UCSC Genome Browser. The BigWig browser track shows a histogram of the read counts and probes are aligned and compared to the read counts of the region they overlap with. Probes that overlapped with more than 5% of the highest read count were used (green) and those that aligned with regions considered as "low read count" (5% or less) were removed from the final probe list (red).*

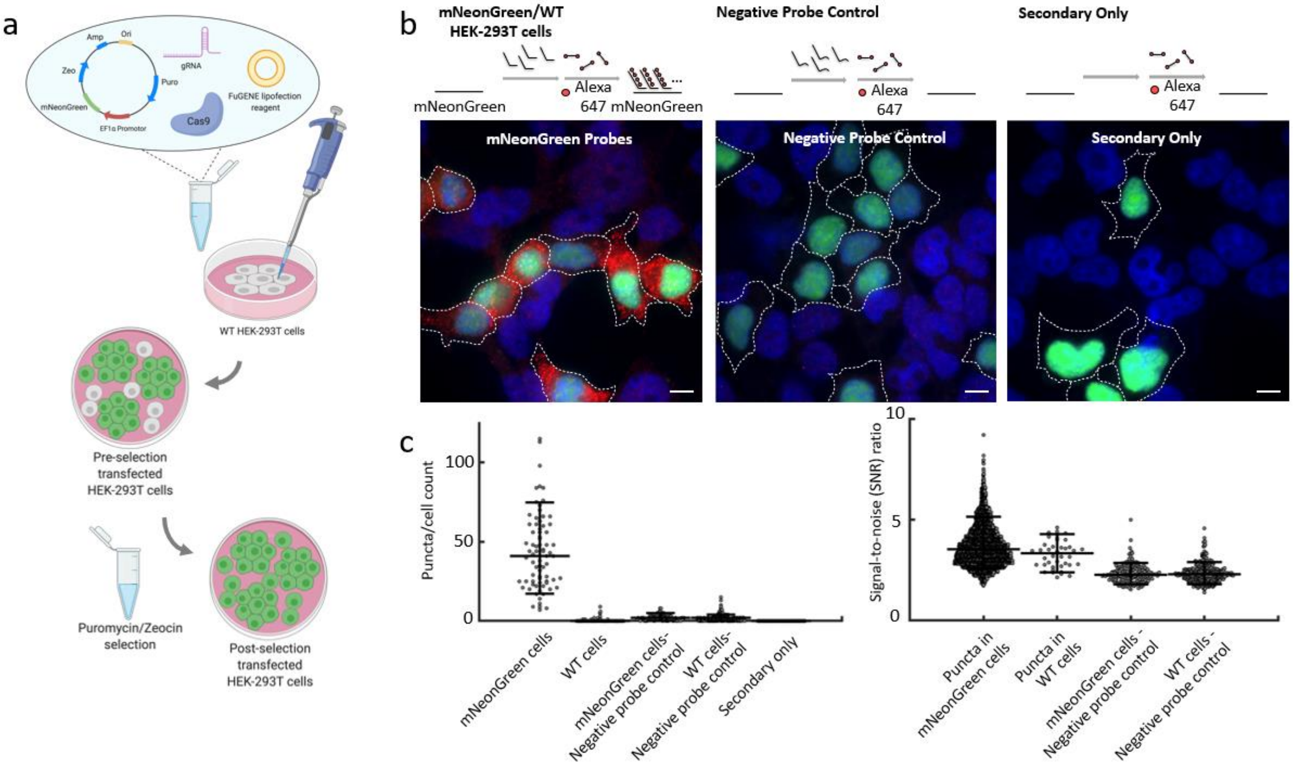

Supplementary Fig. 2. Validation of probe hybridization in mNeonGreen cells. *a) Schematic of engineering of mNeonGreen HEK293T-X cells. Engineered mNeonGreen plasmids were transfected into HEK293T-X cells with FuGENE HD Transfection Reagent. Three days after transfection, the cells were then selected with Puromycin and Zeocin.* ***b)*** *Schematic and representative images of each condition. The primary probes were designed to be complementary to mNeonGreen transcripts. A dopachrome tautomerase (DCT) primary probe negative control, which uses primary probes targeting sequences not present in the mNeonGreen HEK-293T-X cells but can still bind to secondary fluorescent oligonucleotides, was used to indicate any nonspecific binding which can occur with primary probe labeling. A negative control where only secondary probes were added but no primary probes were added was used as a reference for nonspecific binding from secondary probes alone. For each condition, the concentration of each primary probe (14 in total) was 1 nM and the secondary probe was 5 nM. Scale bar = 10 µm.* ***c)*** *Dot plots quantifying the detected puncta per cell and signal-to-noise (SNR) ratio under different conditions. Overlaid lines correspond to quantiles [10 50 90]%. Left, scatter plot showing puncta number per cell (n=755 cells). Right, signal-to-noise ratio (SNR). SNR=each signal intensity/the mean of background noise (n=3,860 puncta). Source data are provided as a Source Data file.*

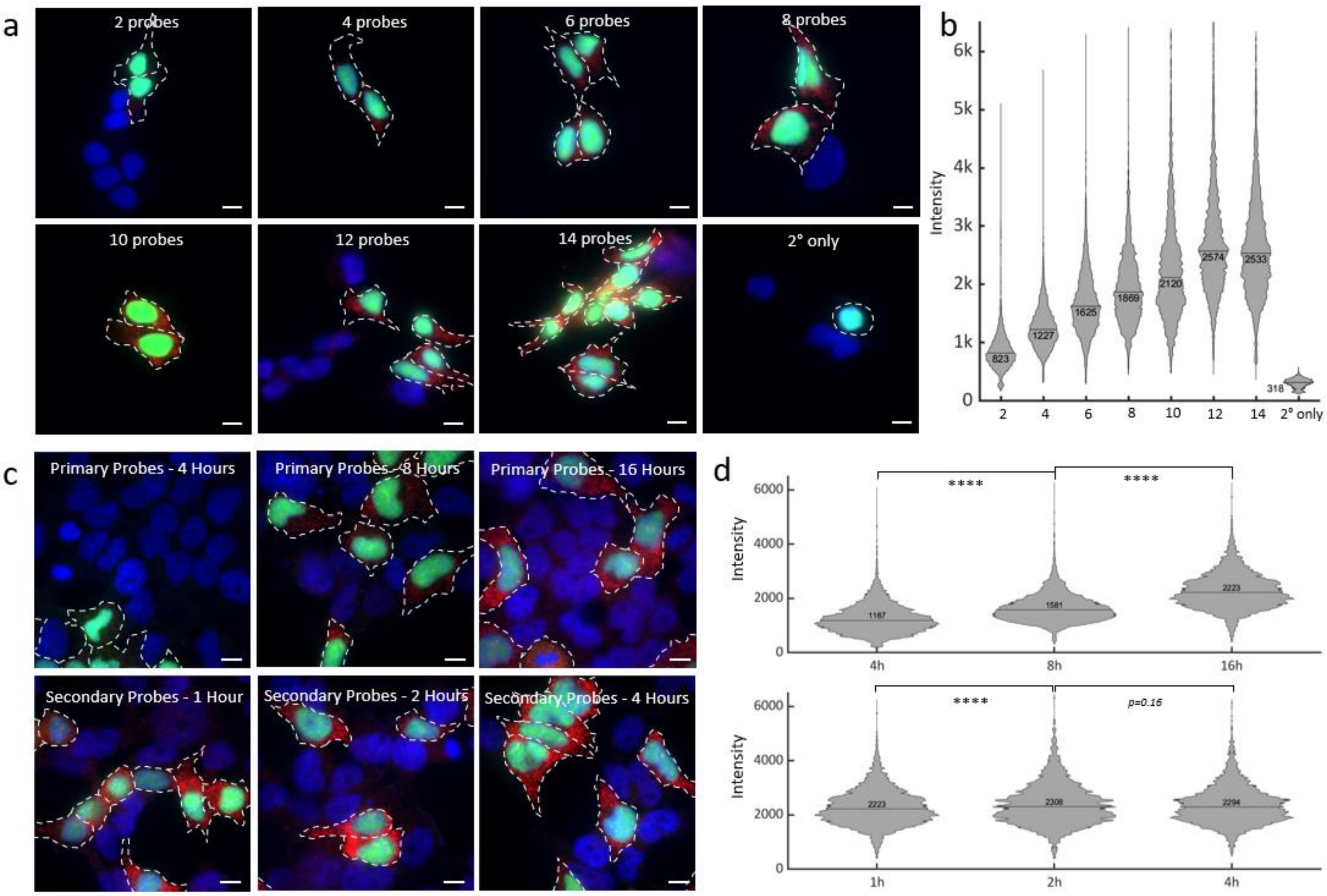

Supplementary Fig. 3. Optimization parameters of in situ hybridization conditions. ***a)*** *Representative images of mNeonGreen cells with different numbers of primary probes. Conditions included 2, 4, 6, 8, 10, 12, and 14 mNeonGreen primary probes to the HEK293T-X mNeonGreen and WT model. The concentration of each primary probe is constant (5 nM). Scale bars are 10 µm.* ***b)*** *Intensity distribution of detected puncta shows the effects of the number of primary probes on signal intensity (total n ≈ 64k puncta).* ***c)*** *Representative images of mNeonGreen cells with different incubation time of probes. The primary probes hybridization incubation times consisted of 4, 8, and 16 hours. For secondary fluorophore probes, incubation times tested were 1, 2, and 4 hours. Scale bars are 10 µm.* ***d)*** *Intensity distribution of detected puncta as a function of incubation time. Top, primary probe incubation time (total n ≈ 26k puncta). Bottom, secondary probe incubation time (total n ≈ 20k puncta). Pairwise t-test (2-tailed) resulted in negligible p-values for conditions marked with **** ($p<10^{-12}$). Source data are provided as a Source Data file.*

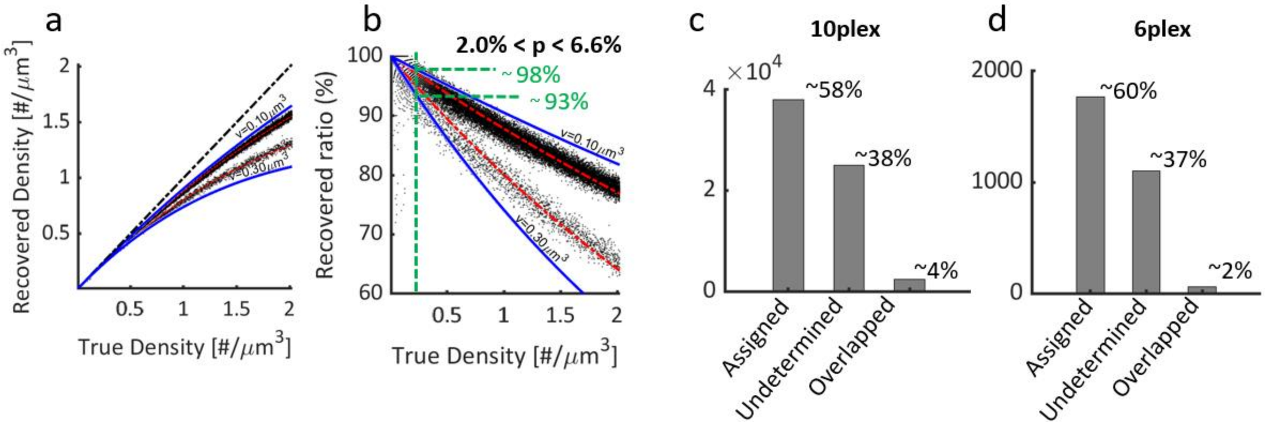

Supplementary Fig. 4. Overlapping and inconsistent signal simulations. *Simulations are run at different densities to generate 3D image stacks and puncta are detected using MOSAICA image processing pipeline.* ***a)*** *Recovered density from the simulations as a function of the true density (top trend for 1plex bottom trend for 10-plex) in blue the computed limits considering the specified puncta volumes.* ***b)*** *Same data plotted as a percentage of recovered puncta. For the 1plex, there cannot be inconsistent signal due to overlap, the underestimated percentage is solely due to undercounts in the segmentation (top trend). For the 10-plex simulation (bottom trend) the recovered loss is also due to inconsistent signal. Again, in blue, the theoretical limits considering extremal puncta volumes. Green vertical line marks the approximate measured density in the experiments and provides an estimation of the loss due to overlap.* ***c, d)*** *Number of puncta assigned to a particular gene, undetermined puncta and overlapped puncta for the 10-plex experiment (Fig. 4 in the paper) and 6-plex experiment (Fig. 5 in the paper). Source data are provided as a Source Data file.*

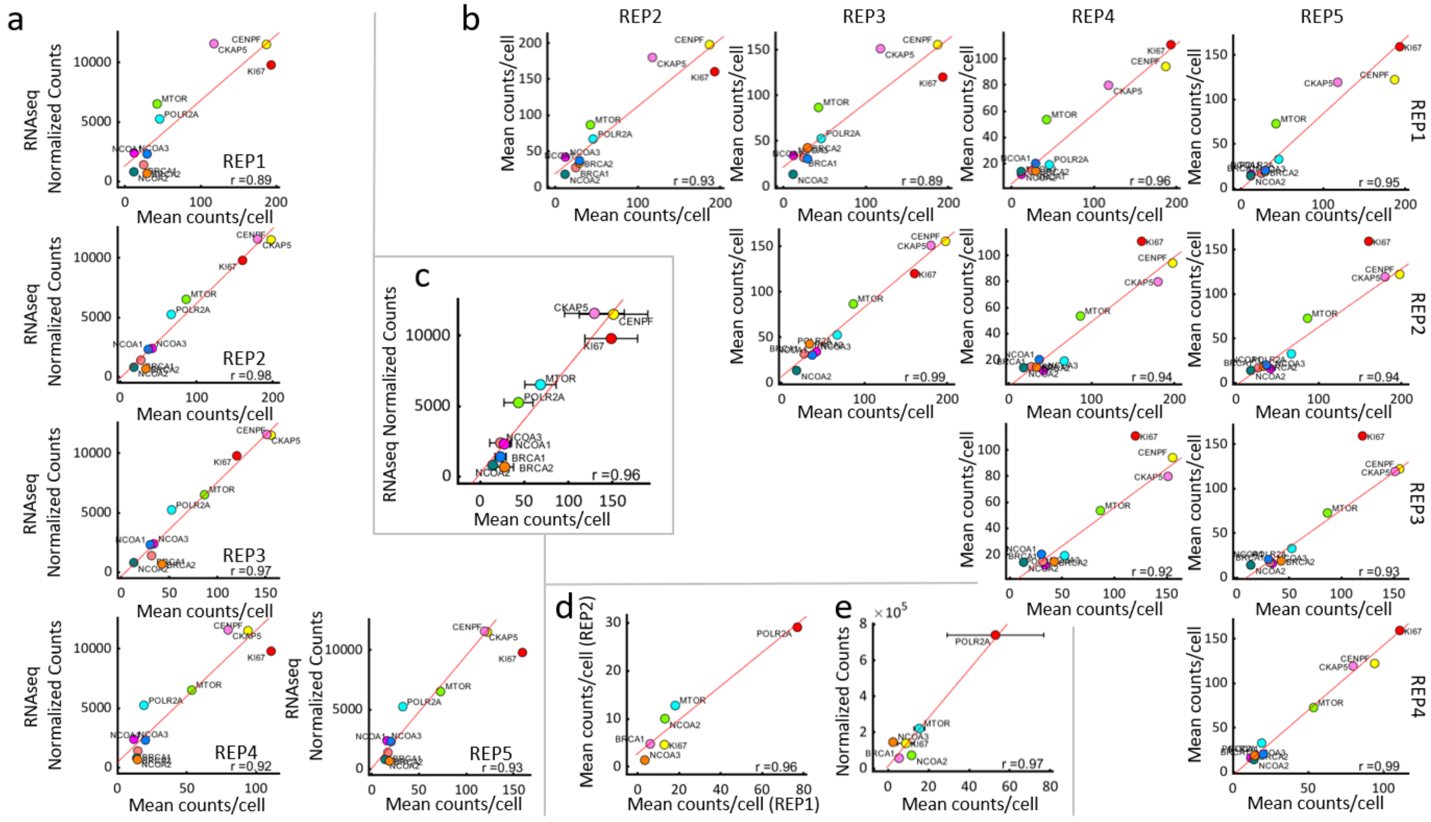

Supplementary Fig. 5. Replicates of the experiments. *A total of 5 cell culture sample replicates for the 10-plex detection of genes in colorectal cancer SW480 cells and a total of 2 experimental replicates for the 6-plex melanoma skin FFPE tissues were imaged.* ***a)*** *Each of the cell replicates is compared to our RNA sequencing counts.* ***b)*** *Cross comparison against each other for the cell culture replicates.* ***c)*** *The averaged values are compared to RNA sequencing in cell culture experiments (n=3 experimental replicates).* ***d)*** *Cross comparison against each other for the tissue replicates.* ***e)*** *Averaged values compared to RNA sequencing in tissue experiments (n=2). Pearson*

correlation coefficient is reported in each case. Where error bars are present, data are presented as mean +/- standard deviation. Source data are provided as a Source Data file.

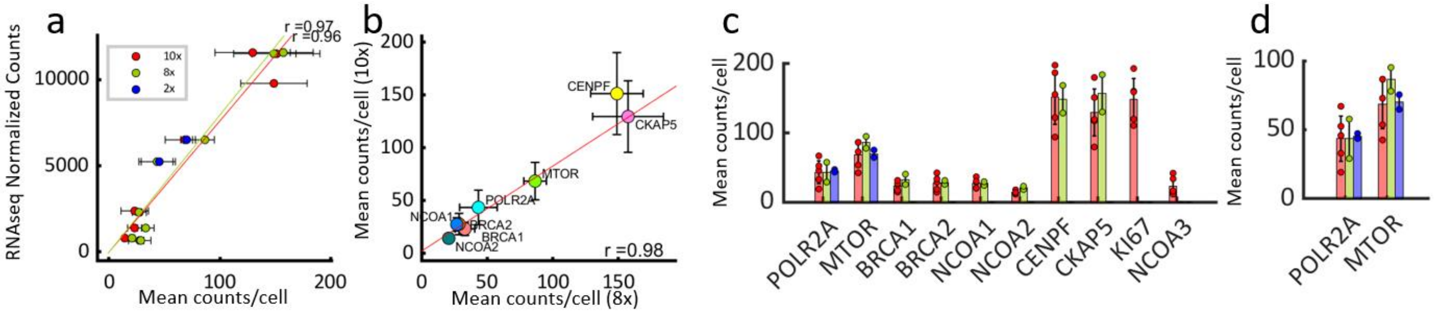

Supplementary Fig. 6. *Assessing detection rates of genes in the 10-plex experiments with 8-plex and 2-plex experiments. For these experiments, the 8-plex panel comprises POLR2A, MTOR, BRCA1, BRCA2, NCOA1, NCOA2, CENPF, and CKAP5. The 2-plex panel comprises POLR2A and MTOR.* ***a****) Mean puncta counts per cell for each gene in the 10-plex, 8-plex and 2-plex experiments against RNA sequencing data. A Pearson correlation of 0.97 and 0.96 for the 10-plex and 8-plex to RNA sequencing data are shown.* ***b****) Mean puncta counts of the 8-plex experiments were correlated with the 10-plex experiments, Pearson r = 0.98.* ***c****) Plotted mean puncta counts per cells for each gene obtained from the 10-plex, 8-plex and 2-plex experiments for comparison.* ***d****) Detail of the two genes tagged in all three sets of experiments. Binomial test between the counts of the 2-plex and expected proportion from the 10-plex gives p=0.0796. Where error bars are present, data are presented as mean +/- standard deviation, n=5 for the 10x, and n=2 for 8x and 2x. Source data are provided as a Source Data file.*

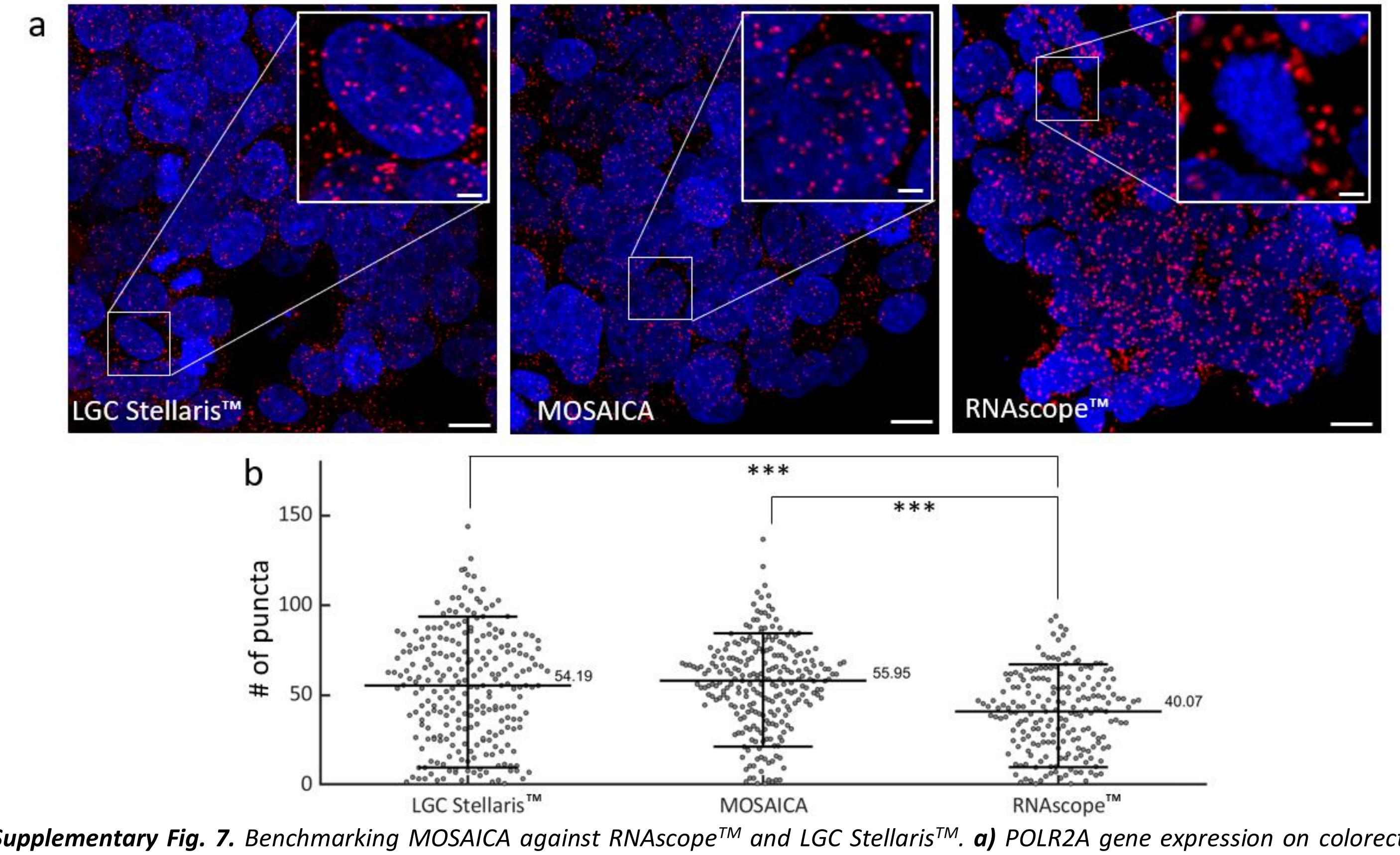

Supplementary Fig. 7. *Benchmarking MOSAICA against RNAscope™ and LGC Stellaris™.* ***a)*** *POLR2A gene expression on colorectal cancer SW480 cells following RNAscope™, LGC Stellaris™ and MOSAICA protocols. Scale bars are 10µm.* ***b)*** *Puncta counts per cell volume between three platforms. Overlaid lines correspond to quantiles [10 50 90]%. MOSAICA exhibited comparable puncta per cell count compared to benchmark LGC Stellaris™, whereas RNAscope™ was undercount. Pairwise t-test (2-tailed) against null hypothesis that values belong to distributions of equal means were p = 0.4 (LGC Stellaris™ vs MOSAICA), p = 3.4x10^{-4} (LGC Stellaris™ vs RNAscope™ and p = 7.8x10^{-4} (MOSAICA vs RNAscope™). A sliding volume of 3000µm^3 was used throughout the image stacks (with an overlap of .25 of its linear dimension) yielding to a total of n=250, n=250 and n=200 volumes respectively. The number of puncta counts per volume was then obtained and divided into the average number of cells per volume depending on the 3D segmentation of DAPI nuclei. Scale bars are 10µm in large images and 2µm in insets. Source data are provided as a Source Data file.*

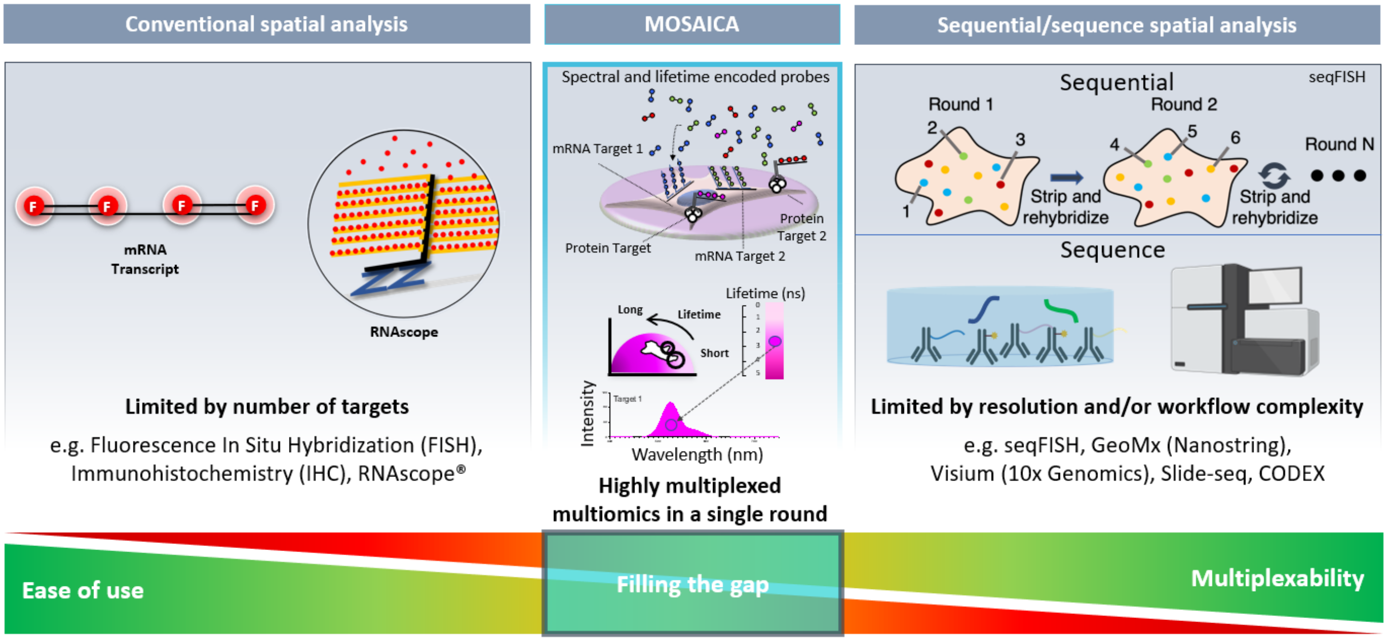

Supplementary Fig. 8. *Multiplexed spatial transcriptomics with MOSAICA, which is rapid, cost-effective, and easy-to-use, can fill a critical gap between conventional FISH and sequential- and sequencing-based techniques.*

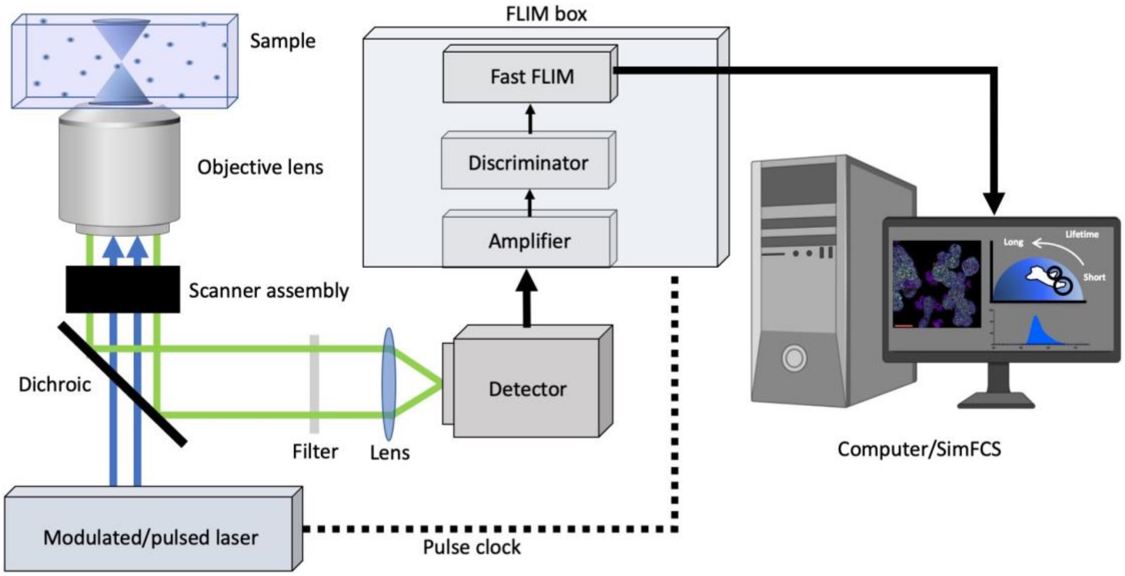

Supplementary Fig. 9. Generalized spectral-FLIM Microscopy setup*. A pulsed/modulated light source is used to illuminate the sample and the fluorescence of the sample is collected by a spectral detector (current resolution around 10 nm). The repetition rate can either be supplied by or delivered to the laser which is used by the electronics in the digital frequency domain to obtain a single photon arrival time using the heterodyne principle (current resolution around 50ps)*

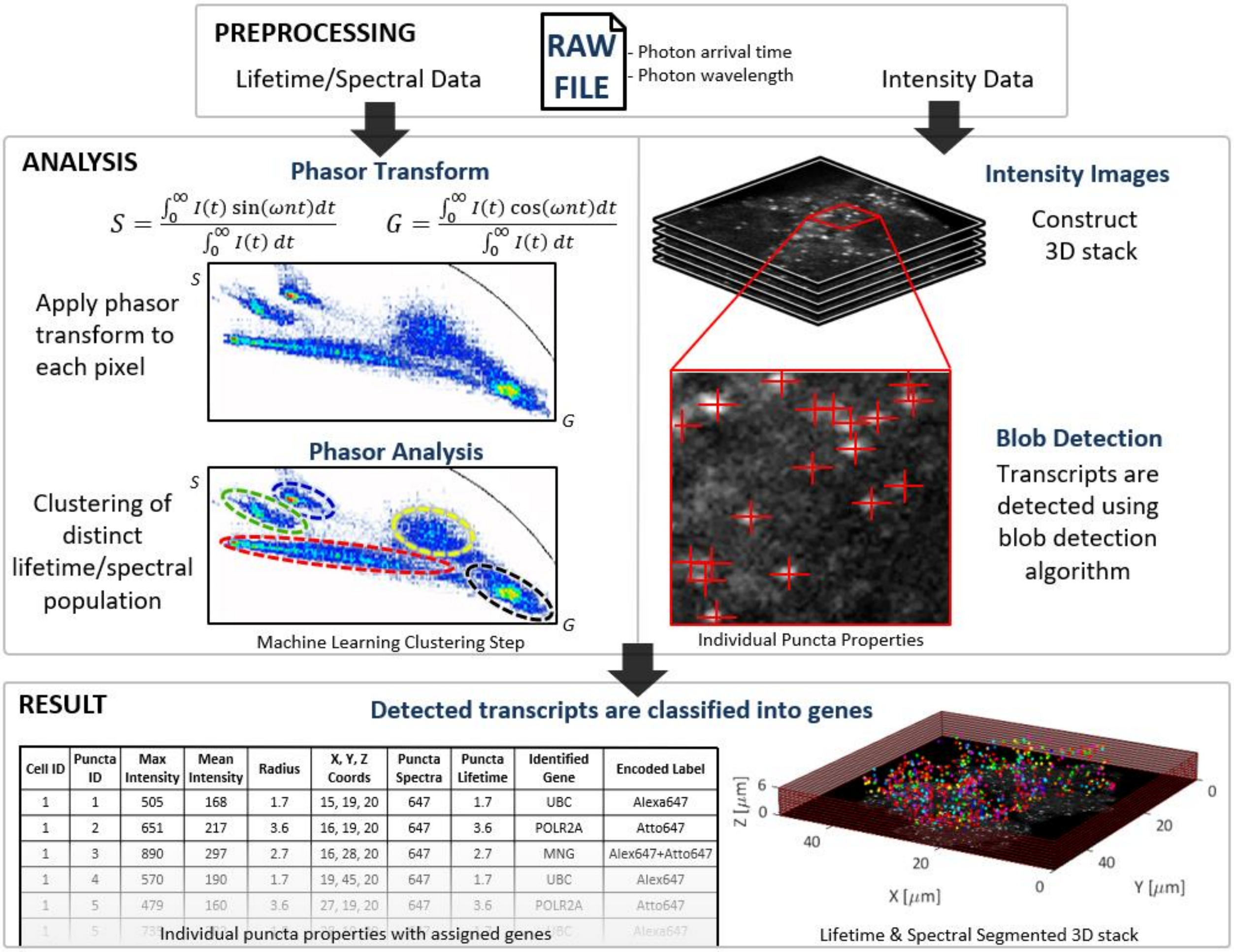

Cell ID	Puncta ID	Max Intensity	Mean Intensity	Radius	X, Y, Z Coords	Puncta Spectra	Puncta Lifetime	Identified Gene	Encoded Label
1	1	505	168	1.7	15, 19, 20	647	1.7	UBC	Alexa647
1	2	651	217	3.6	16, 19, 20	647	3.6	POLR2A	Atto647
1	3	890	297	2.7	16, 28, 20	647	2.7	MNG	Alex647+Atto647
1	4	570	190	1.7	19, 45, 20	647	1.7	UBC	Alex647
1	5	479	160	3.6	27, 19, 20	647	3.6	POLR2A	Atto647
1	5	[illegible]	[illegible]	[illegible]	[illegible]	[illegible]	[illegible]	UBC	Alexa647

Supplementary Fig. 10. Automated pipeline of the processing and analysis. *Raw data consists of a list of detected photons with their times of arrivals. Using the acquisition parameters, dwell time, number of pixels, number of repetitions per image etc. the image stacks are reconstructed. Knowing the laser frequency, a photon histogram for each voxel is built and the phasor transform is applied. The two custom made algorithms work in parallel, one identifying clusters in the phasor space, the other identifying puncta in the intensity space. The two then recombine to result in each transcript being identified, assigned to a particular gene and its morphological properties measured.*
