# Supplementary material for: Spatial transcriptomics using combinatorial fluorescence spectral and lifetime encoding, imaging and analysis
Source: Nat Commun. 2022 Jan 10;13:169. doi: 10.1038/s41467-021-27798-0 (PMC8748653; doi:10.1038/s41467-021-27798-0)
Supplement: Supplementary file 3 — Description of Additional Supplementary Files [file 41467_2021_27798_MOESM3_ESM.pdf]

File Name: Supplementary Data 1

Description: List of probe sequences used for each target gene in this study comprising the "full sequence" of the probes as well as sequence regions that bind to the target ("Target Region") and secondary probes ("Readout 1" and "Readout 2"). See "Supplementary Data 1" xls file.
