# Supplementary material for: Spatial transcriptomics using combinatorial fluorescence spectral and lifetime encoding, imaging and analysis
Source: Nat Commun. 2022 Jan 10;13:169. doi: 10.1038/s41467-021-27798-0 (PMC8748653; doi:10.1038/s41467-021-27798-0)
Supplement: Supplementary file 5 — Reporting Summary [file 41467_2021_27798_MOESM5_ESM.pdf]

# nature portfolio

Corresponding author(s): Weian Zhao and Enrico Gratton

Last updated by author(s): Dec 3, 2021

# Reporting Summary

Nature Portfolio wishes to improve the reproducibility of the work that we publish. This form provides structure for consistency and transparency in reporting. For further information on Nature Portfolio policies, see our Editorial Policies and the Editorial Policy Checklist.

## Statistics

For all statistical analyses, confirm that the following items are present in the figure legend, table legend, main text, or Methods section.

| n/a | Confirmed | |
|---|---|---|
| ☐ | ☒ | The exact sample size ($n$) for each experimental group/condition, given as a discrete number and unit of measurement |
| ☐ | ☒ | A statement on whether measurements were taken from distinct samples or whether the same sample was measured repeatedly |
| ☐ | ☒ | The statistical test(s) used AND whether they are one- or two-sided<br>*Only common tests should be described solely by name; describe more complex techniques in the Methods section.* |
| ☐ | ☒ | A description of all covariates tested |
| ☐ | ☒ | A description of any assumptions or corrections, such as tests of normality and adjustment for multiple comparisons |
| ☐ | ☒ | A full description of the statistical parameters including central tendency (e.g. means) or other basic estimates (e.g. regression coefficient) AND variation (e.g. standard deviation) or associated estimates of uncertainty (e.g. confidence intervals) |
| ☐ | ☒ | For null hypothesis testing, the test statistic (e.g. $F$, $t$, $r$) with confidence intervals, effect sizes, degrees of freedom and $P$ value noted<br>*Give P values as exact values whenever suitable.* |
| ☒ | ☐ | For Bayesian analysis, information on the choice of priors and Markov chain Monte Carlo settings |
| ☒ | ☐ | For hierarchical and complex designs, identification of the appropriate level for tests and full reporting of outcomes |
| ☐ | ☒ | Estimates of effect sizes (e.g. Cohen's $d$, Pearson's $r$), indicating how they were calculated |

*Our web collection on statistics for biologists contains articles on many of the points above.*

## Software and code

Policy information about availability of computer code

| | |
|---|---|
| Data collection | Lifetime microscopy images where acquired using two commercial microscopes with their respective software suites, Leica LASX-FLIM/FCS (version 3.5.6) for the Leica-Falcon microscope and VistaVision (version 4.2.147) for the ISS-ALBA5 microscope. |
| Data analysis | Image processing pipeline developed for this paper has been published to repository Figshare: https://doi.org/10.6084/m9.figshare.14810820.v4 |

For manuscripts utilizing custom algorithms or software that are central to the research but not yet described in published literature, software must be made available to editors and reviewers. We strongly encourage code deposition in a community repository (e.g. GitHub). See the Nature Portfolio guidelines for submitting code & software for further information.

## Data

Policy information about availability of data

All manuscripts must include a data availability statement. This statement should provide the following information, where applicable:

- Accession codes, unique identifiers, or web links for publicly available datasets
- A description of any restrictions on data availability
- For clinical datasets or third party data, please ensure that the statement adheres to our policy

Raw images and data used for Fig. 3, 4, 5, and 6 and Supplementary Figs 2-7 have been uploaded to public repository Figshare (https://doi.org/10.6084/m9.figshare.17072390.v4). A working example (used in Fig. 4) is included with the software package in the Code Availability section. Probe sequences used for labeling are included in the supplementary material section. RNA sequencing data was obtained from publicly available data from The Cancer Genome Atlas (TCGA), available on the National Cancer Institute (NCI) Genomic Data Commons (GDC) data portal (https://gdc.cancer.gov/). Please refer to Sequencing Data section for Entity IDs.

# Field-specific reporting

Please select the one below that is the best fit for your research. If you are not sure, read the appropriate sections before making your selection.

☒ Life sciences ☐ Behavioural & social sciences ☐ Ecological, evolutionary & environmental sciences

For a reference copy of the document with all sections, see nature.com/documents/nr-reporting-summary-flat.pdf

# Life sciences study design

All studies must disclose on these points even when the disclosure is negative.

| | |
|---|---|
| Sample size | Sample size was measured in terms of number of cells profiled for each experimental condition.<br>For experiments involving detection and quantification of puncta, sample sizes were defined as the number of cells which were profiled for its corresponding gene panel. 364 cells were profiled for the 10 plex plus 189 cells for the negative probe controls in the cell experiments. For the 6-plex tissue experiments, 174 cells were profiled for the 6-plex experiments and 375 cells for the negative probe controls. Over 750 cells were profiled in the supplementary probe labeling and validation experiments (8 plex and 2 plex experiments). We determined that these samples sizes were sufficient to profile transcript abundance of targeted genes from cells consistently across replicates. These sample sizes are generally comparable to those used in the literature for initial spatialomics tool development. Comparison was done on a transcript basis for each condition with matching controls. |
| Data exclusions | No data were excluded for the cell culture images. In the tissue field of view, the outermost layer of the epidermis section containing no cells was excluded in order to analyze only the relevant volume of tissue containing cells. |
| Replication | All our replicates were successful and the results were consistent.<br>For SW480 10-plex experiment, a total of n=3 experimental replicates were performed including controls for each were profiled. For skin FFPE tissue experiment, a total of of two experimental replicates were performed including controls. Additional controls were performed for the SW480 cell profiling in which n=2 experimental replicates were prepared with only 8 out of the 10 targets, and another 2 experimental replicates with only 2 out of the 10 targets. For benchmarking experiments, at least 2 samples were used for each assay. For mNeon green cell samples, 24 samples were analyzed. |
| Randomization | For cell experiments, imaged regions for analysis were selected randomly while for tissue experiments, imaged sections were selected to target and profile the epidermis region of the samples where the target molecules are located. |
| Blinding | Studies were not blinded as experimental conditions were selected to confirm and validate assay conditions and not to verify any clinical condition or treatment. Analysis of each experimental condition was performed with the same sets of parameters in the image processing scripts. |

# Reporting for specific materials, systems and methods

We require information from authors about some types of materials, experimental systems and methods used in many studies. Here, indicate whether each material, system or method listed is relevant to your study. If you are not sure if a list item applies to your research, read the appropriate section before selecting a response.

## Materials & experimental systems

| n/a | Involved in the study |
|---|---|
| ☐ | ☒ Antibodies |
| ☐ | ☒ Eukaryotic cell lines |
| ☒ | ☐ Palaeontology and archaeology |
| ☒ | ☐ Animals and other organisms |
| ☐ | ☒ Human research participants |
| ☒ | ☐ Clinical data |
| ☒ | ☐ Dual use research of concern |

## Methods

| n/a | Involved in the study |
|---|---|
| ☒ | ☐ ChIP-seq |
| ☒ | ☐ Flow cytometry |
| ☒ | ☐ MRI-based neuroimaging |

## Antibodies

| | |
|---|---|
| Antibodies used | 1) Rabbit anti-Vimentin. Vendor: Cell Signaling. Clone: D21H3. Catalog #: 5741BF. Lot #: 8. 2) Mouse anti-alpha Tubulin. Vendor: Cell Signaling. Clone: DM1A. Catalog #: 3873BF. Lot #: 16. 3) Donkey anti-Mouse Alexa-488. Vendor: Fisher Scientific. Clone: Polyclonal. Catalog #: R37114. Lot # 2303213. 4) Donkey anti-Rabbit TRITC. Vendor: Jackson Laboratories. Clone: Polyclonal. Catalog #: 711-025-152. Lot #: 156613. |
| Validation | Antibodies 1 and 2 were validated for immunofluorescence and immunohistochemistry applications using cell lines and paraffin-embedded tissues and have met all of the quality control standards defined by Cell Signaling as stated in their certificate of analysis documents which can be found on their product links on cellsignal.com. Antibody 3 has been tested and meets the quality standards of Molecular Probes as stated in the product's Certificate of Analysis by lot. For immunocytochemistry applications, this product has |

been cited in 5 publications which can be found on the product link from thermofisher.com. Antibody 4 has been validated for its antibody specificity via immunoelectrophoresis and/or ELISA to react with whole molecule rabbit IgG and for its minimal cross-reaction to non-rabbit species IgG as stated in the product specification sheet which can be found on the product link on jacksonimmuno.com. CiteAb.com provides 4 publications which have utilized this product for immunocytochemistry applications.

## Eukaryotic cell lines

Policy information about cell lines

| | |
|---|---|
| Cell line source(s) | 1) SW480 cells from ATCC CCL-228. 2) Lenti-X HEK 293T Cell Line from Takara Biosciences 632180. |
| Authentication | Cell line has been authenticated by ATCC by STR profiling while cell line 2 has not been authenticated. |
| Mycoplasma contamination | Both cells lines have tested negative for mycoplasma contamination by the vendor. Mycoplasma contamination tests were not routinely performed subsequently because no indication of aberrant growth or behavior was noticed. |
| Commonly misidentified lines (See ICLAC register) | No commonly misidentified lines were used. |

## Human research participants

Policy information about studies involving human research participants

| | |
|---|---|
| Population characteristics | N/A |
| Recruitment | N/A |
| Ethics oversight | The University of California, Irvine IRB approved this study for IRB exemption under protocol number HS# 2019-5054 |

Note that full information on the approval of the study protocol must also be provided in the manuscript.
